# A rare case of inflammatory myofibroblast tumor of the stomach successfully treated by inverted laparoscopic and endoscopic cooperative surgery

**DOI:** 10.1186/s40792-023-01767-9

**Published:** 2023-10-30

**Authors:** Naoya Kimura, Masatsugu Hiraki, Michiaki Akashi, Koichi Miyahara, Minori Imamura, Shunsuke Furukawa, Ryuichiro Samejima

**Affiliations:** 1 Department of Surgery, Japan Red Cross Society Karatsu Red Cross Hospital, 2430 Watada, Karatsu, Saga 847-8588 Japan; 2 Department of Pathology, Japan Red Cross Society Karatsu Red Cross Hospital, Karatsu, Saga Japan; 3 Department of Internal Medicine, Japan Red Cross Society Karatsu Red Cross Hospital, Karatsu, Saga Japan

**Keywords:** Inflammatory myofibroblast tumor, Stomach, Submucosal tumor, Laparoscopic and endoscopic cooperative surgery

## Abstract

**Background:**

An Inflammatory myofibroblastic tumor (IMT) is a rare intermediate malignancy characterized by myofibroblast proliferation and inflammatory cell infiltration. Various organs are the primary sites of origin. However, primary tumors originating in the stomach tend to be extremely rare, making the diagnosis difficult. Herein, we present a case of IMT originating in the stomach that was effectively managed using inverted laparoscopic endoscopic cooperative surgery (LECS).

**Case presentation:**

A 47-year-old male who was admitted to the hospital because of a submucosal tumor that was discovered during upper gastrointestinal endoscopy. The diameter of the tumor was approximately 20 mm. A KIT-negative gastrointestinal stromal tumor was suspected based on the biopsy findings. Therefore, partial resection of the stomach was performed using inverted laparoscopic and endoscopic cooperative surgery. Histopathological examination revealed collagen fiber proliferation from the submucosal layer to the muscular layer, accompanied by infiltration of spindle-shaped cells, lymphocytes, and numerous inflammatory cells. Immunohistochemistry results were positive for SMA and negative for CD34, desmin, and c-kit. IgG4-positive cells were observed with an IgG4/IgG ratio > 50%, and specific nuclei were positive for ALK. Therefore, IMT was diagnosed. This condition may be difficult to diagnose both before and after surgery because of its rarity and submucosal tumor-like morphology.

**Conclusion:**

When a submucosal tumor originating in the stomach is observed, IMT should be considered. Partial resection of the stomach with LECS and immunohistochemical diagnosis may be useful.

## Background

Inflammatory myofibroblastic tumor (IMT) is an intermediate malignancy characterized by the proliferation of myofibroblasts and infiltration of inflammatory cells such as lymphocytes, plasma cells, and eosinophils [1]. Although the most common site of occurrence is the lung, IMTs have also been reported to originate in the greater omentum, mesentery, retroperitoneum, genitourinary system, central nervous system, and musculoskeletal system [2, 3]. Symptoms of IMT include fever, anemia, and weight loss. However, there were no specific clinical features. IMTs are often discovered incidentally and there are no specific laboratory findings. We report a case of IMT originating in the stomach that was successfully treated with inverted laparoscopic endoscopic cooperative surgery (LECS).

## Case presentation

**Fig. 1:**
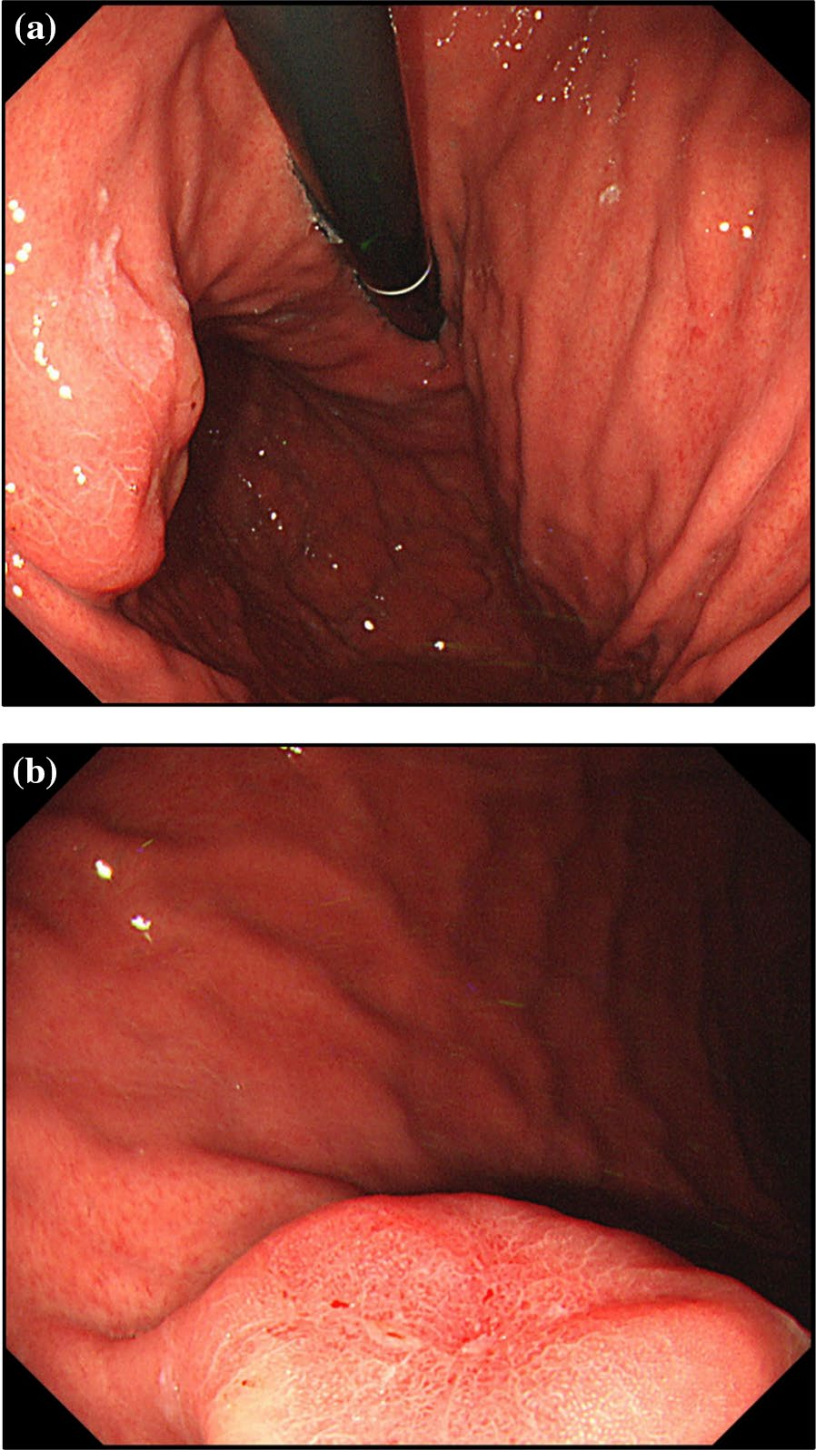
A submucosal tumor measuring approximately 20 mm in diameter was found on the posterior wall of the middle part of the stomach (**a**). A depression on the mucosal surface due to a previous biopsy was observed (**b**)

A 47-year-old male was admitted to our hospital because of a submucosal tumor in the middle of the gastric body that was detected during upper gastrointestinal endoscopy performed for screening. There were no notable physical findings and the patient had no relevant medical history. Blood test results revealed no significant abnormalities. Upper gastrointestinal endoscopy and contrast imaging showed a submucosal tumor of 20 mm in diameter on the posterior wall of the gastric body (Fig. 1a). Because of previous biopsy, a depression on the mucosal surface was observed (Fig. 1b). Computed tomography demonstrated slight thickening of the gastric wall of the tumor and no distant metastasis. Endoscopic ultrasound revealed a lesion with an uneven internal echo image originating from the muscle layer. Examination of biopsy specimens obtained by endoscopic ultrasound-guided fine-needle aspiration revealed spindle-shaped cells with fibroblast-like nuclei in tumor cells. Immunohistochemistry showed partial positivity for CD34 and negativity for c-kit. Based on these findings, KIT-negative gastrointestinal stromal tumor (GIST) was suspected. Therefore, a surgical resection was performed.

**Fig. 2:**
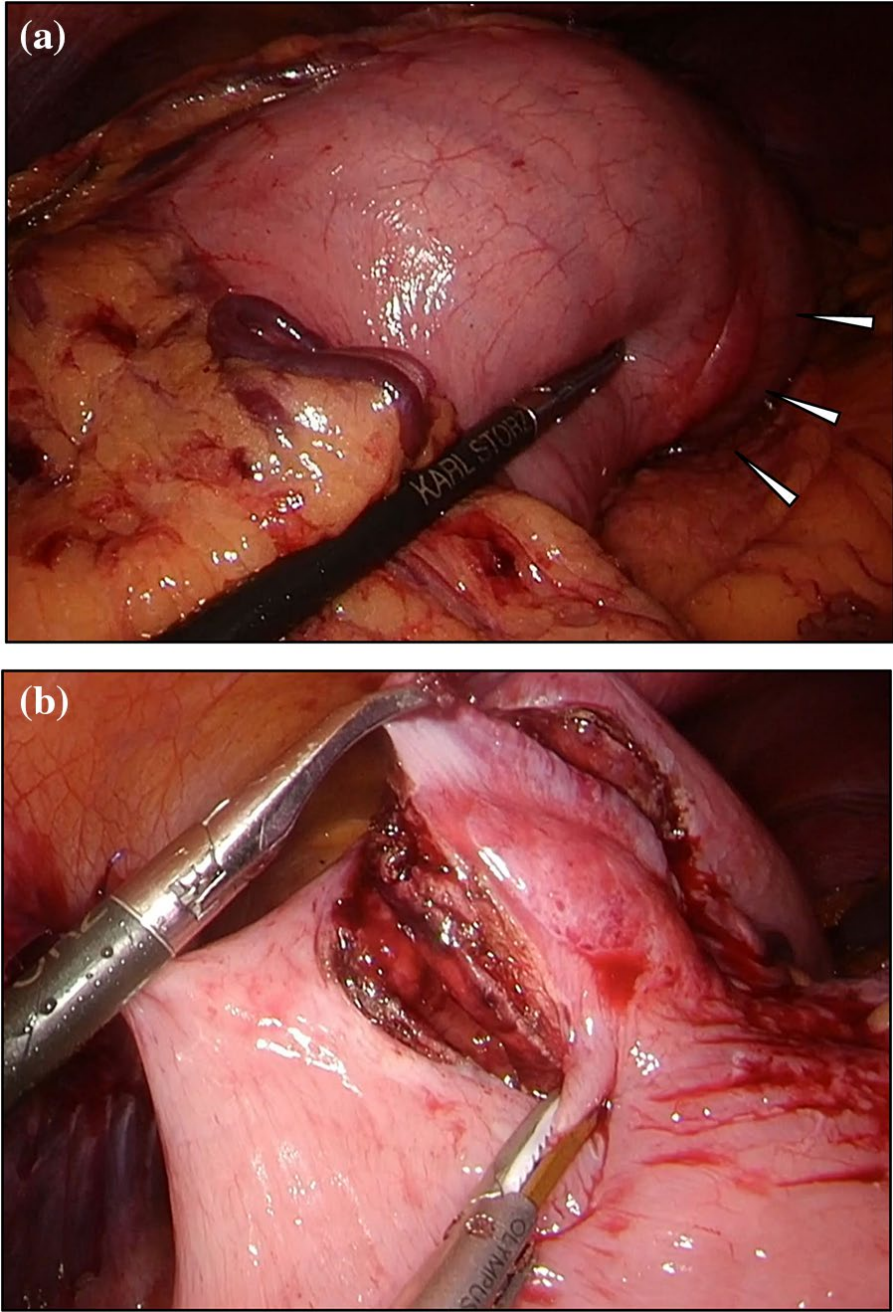
The tumor was located on the posterior wall of the middle part of the stomach and appeared as a reddish-white mass (**a** white arrowhead). The tumor was excised along the dissection line from the extragastric wall under laparoscopic view (**b**)

Intraoperative findings showed a tumor lesion with a white color change on the serosal surface at the posterior wall of the middle part of the stomach after dividing the greater omentum (Fig. 2a). After suturing the stomach wall to the abdominal wall and suspending the stomach, marking was performed on the external wall of the stomach was marked along a line that secured a sufficient margin from the tumor. Endoscopic submucosal dissection was performed from the inside of the stomach along the marking line; when the dissection had progressed to a certain extent, the tumor was excised along the dissection line from the extragastric wall under a laparoscopic view (Fig. 2b). The excised specimen was endoscopically removed from the oral cavity. Closure was performed using an automatic suturing device after suturing the stomach wall with a support thread. After closing the stomach wall, absence of bleeding and deformation was confirmed endoscopically. The operation time was 3 h and 26 min, and there was little blood loss.

**Fig. 3:**
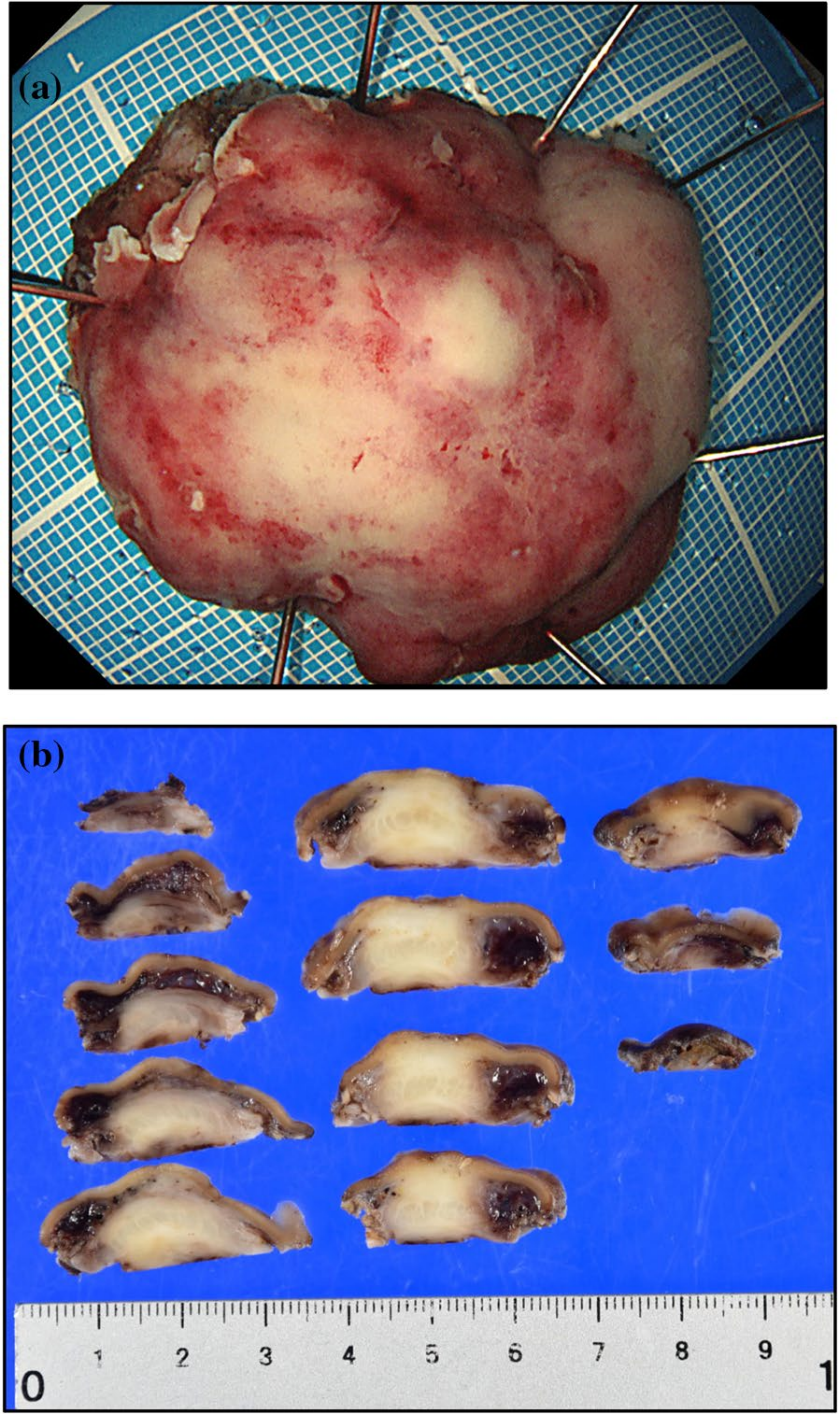
The resected specimen measured 36 × 32 mm (**a**) and showed a gray-white tumorous lesion with a slightly indistinct border measuring approximately 27 × 17 × 11 mm from the submucosal layer to the seromuscular layer on the cut surface (**b**)

**Fig. 4:**
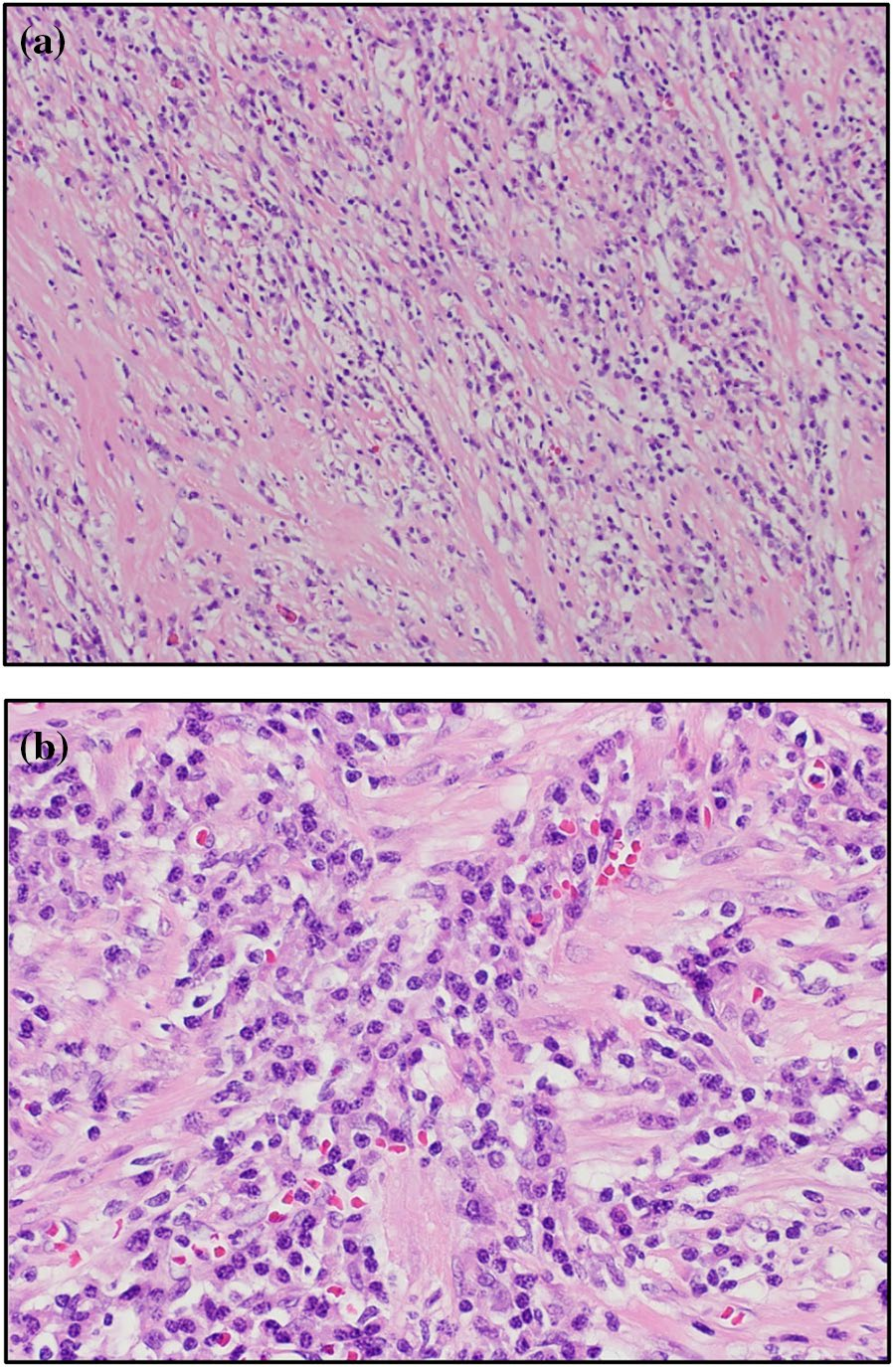
A histopathological examination revealed increased collagen fiber proliferation and the infiltration of spindle-shaped cells, lymphocytes, and numerous stromal cells from the submucosal layer to the muscular layer (**a** hematoxylin and eosin staining, ×40. **b** Hematoxylin and eosin staining, ×200). The mitotic index was 5%

**Fig. 5:**
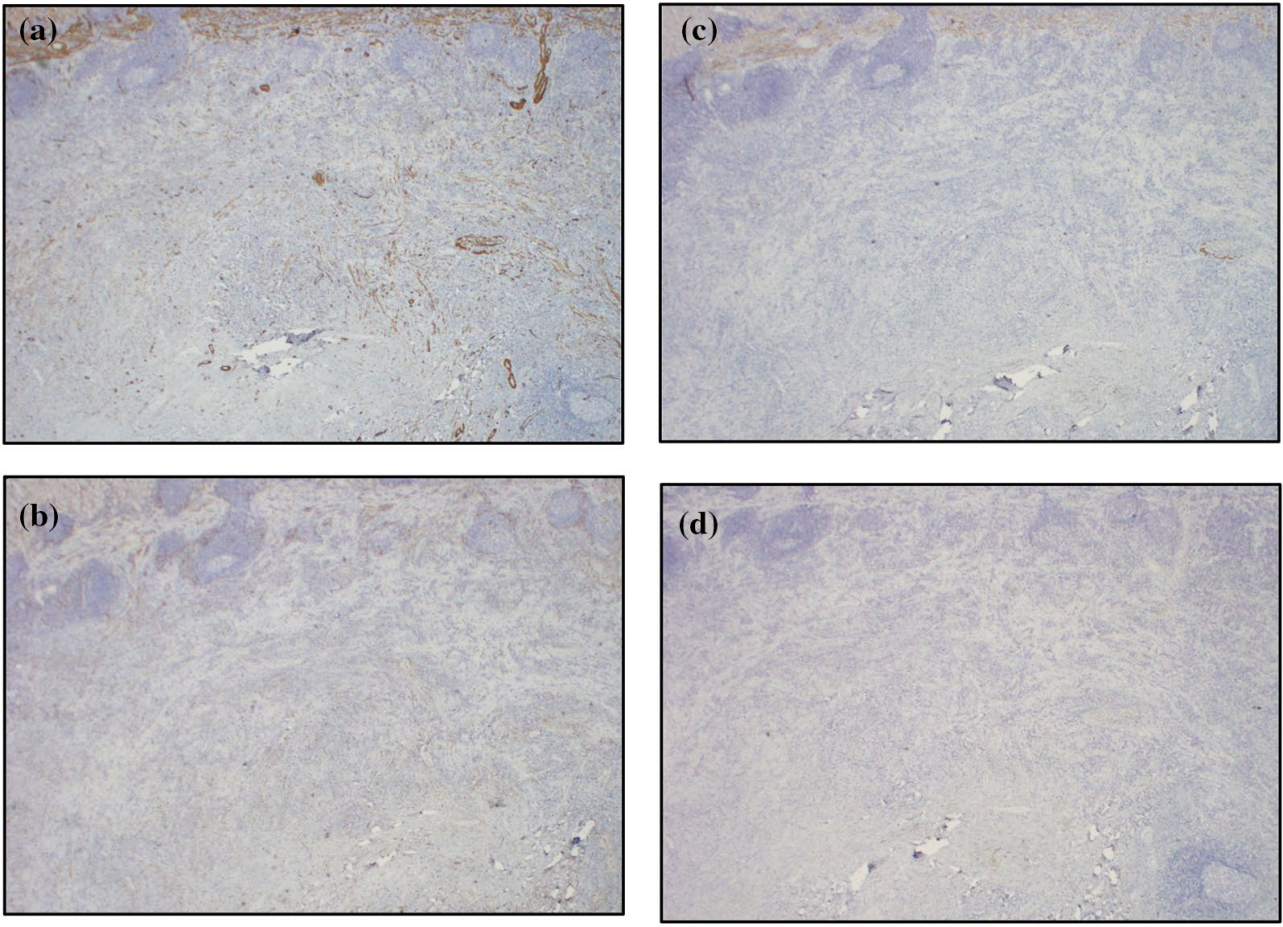
Immunostaining of spindle-shaped tumor cells was positive for SMA (**a**) and negative for CD34 (**b**), desmin (**c**), and c-kit (**d**)

**Fig. 6:**
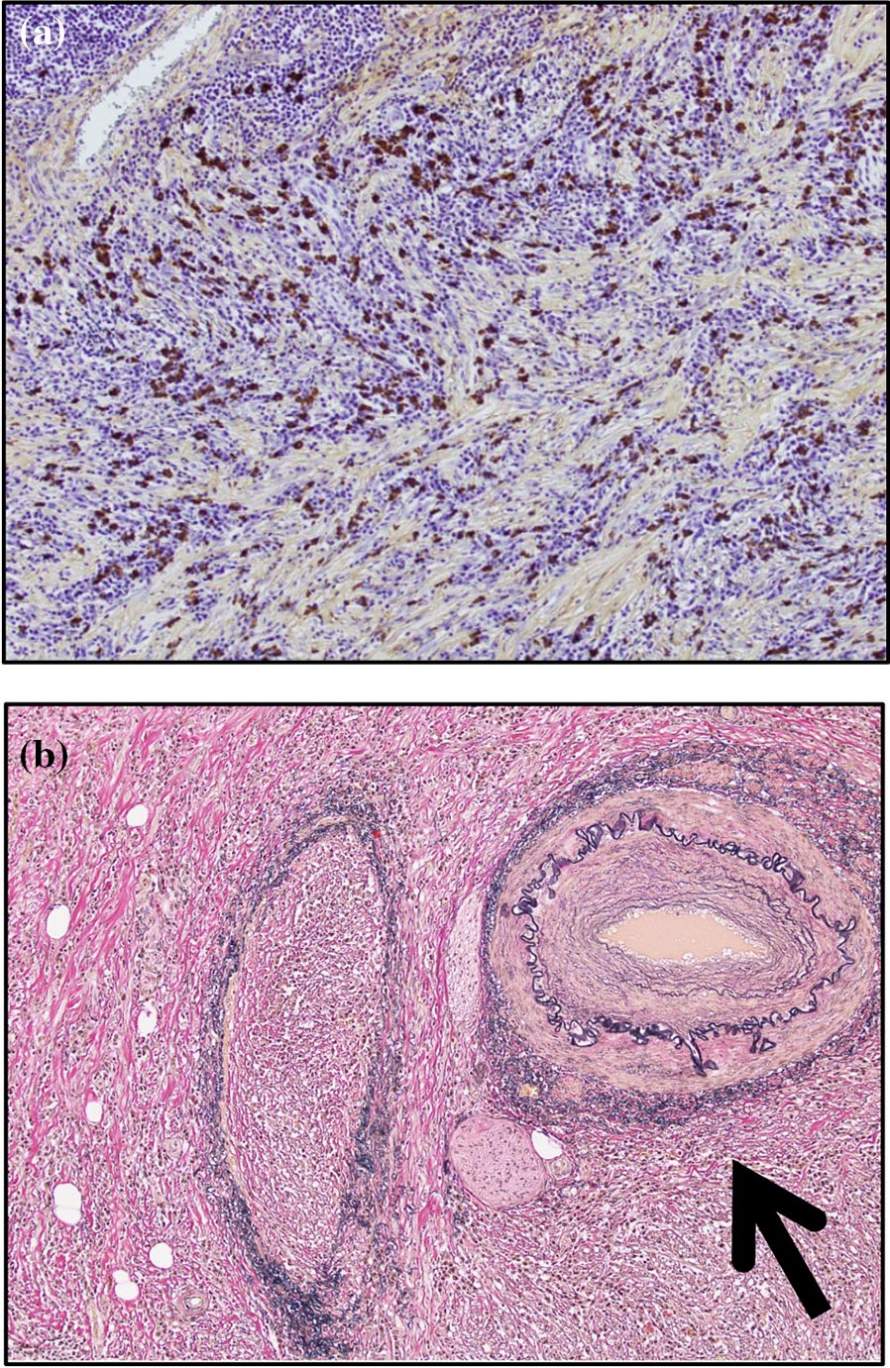
IgG4-positive cells were detected in hot spots at a density of 267/HPF (**a**). EVG staining revealed obstructive phlebitis in two venules (**b** black arrow)

**Fig. 7:**
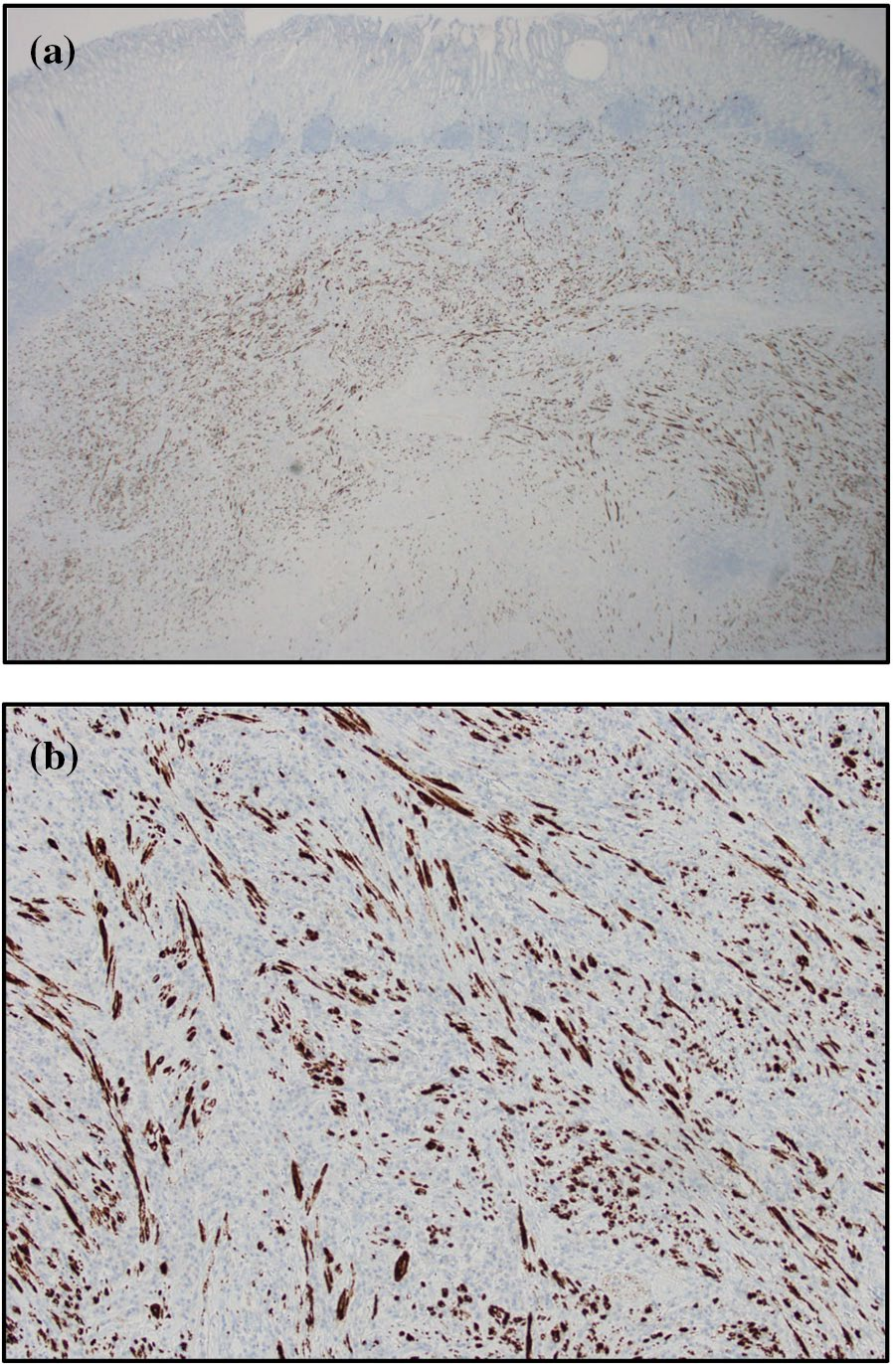
ALK was specifically positive in the nuclei of spindle-shaped cells (**a** ×20, **b** ×200)

**Fig. 8:**
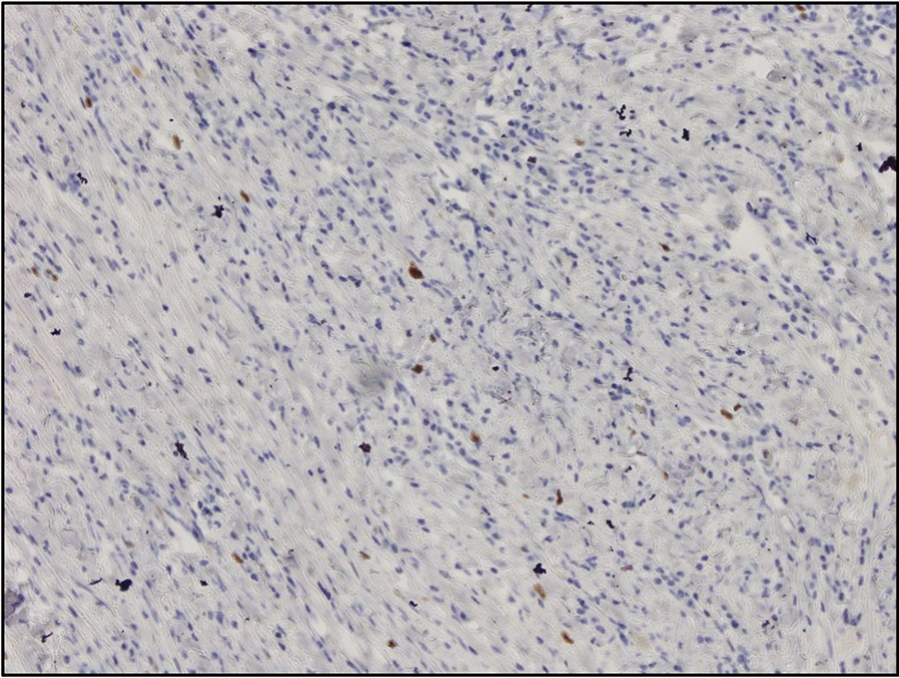
Immunostaining was partially positive for Ki-67, and the MIB-1 index was 2–3% (×10)

The resected specimen measured 36 mm × 32 mm with a sufficient margin (Fig. 3a). A gray-white tumor measuring 27 × 17 × 11 mm was observed from the submucosa to the serosa (Fig. 3b). Histopathological examination revealed proliferation of collagen fibers from the submucosal layer to the muscular layer, accompanied by the infiltration of spindle-shaped cells, lymphocytes, and numerous inflammatory cells (Fig. 4). Immunohistochemistry showed that the nuclei of spindle-shaped tumor cells were positive for SMA (Fig. 5a) and negative for CD34, desmin, and c-Kit (Fig. 5b–d). Additionally, IgG4-positive cells were observed (Fig. 6a) with an IgG4/IgG ratio of > 50%. Elastica van Gieson (EVG) staining revealed obstructive phlebitis in two venules (Fig. 6b). An additional immunohistochemical study revealed positivity for IgG4 and a specific nuclear positivity for ALK (Fig. 7). The mitotic index was 5% (Fig. 4), and immunostaining of Ki-67 was partially positive, while the MIB-1 index was 2–3% (Fig. 8). Therefore, in addition to the absence of an increase in serum IgG4 levels, a diagnosis of inflammatory myofibroblastic tumor (IMT) was made. The patient was discharged on the eighth day after surgery and showed no signs of recurrence during the follow-up period of 1 year and 6 months after the operation.

## Discussion

IMT is characterized by the proliferation of spindle-shaped cells, typical of myofibroblasts, along with infiltration of inflammatory cells such as lymphocytes and plasma cells [1]. IMTs most commonly occurred in the lungs (22%). However, they have also been reported in the head and neck, chest wall, digestive tract, urinary tract, and soft tissue of bones [2, 3]. In particular, reports of primary IMTs in the stomach of adults are rare. A search of the PubMed database using the keywords "stomach" and "inflammatory myofibroblast tumor" for articles published until March 2023 identified 37 cases of primary IMT of the stomach in adults, including the current case (Table 1) [4–29]. The median age of the patients was 42 years; 57% (21/37) of the cases occurred in women and 43% (16/37) occurred in men. Surgical resection and endoscopic treatment were performed in 95% (35/37) and 5% (2/37) of the cases, respectively. Among patients who underwent surgical resection, 5.7% (2/35) underwent combined surgery with endoscopy. None of the cases were diagnosed preoperatively, indicating the difficulty of diagnosing IMT before surgery.

**Table 1:** Summary of previous reports on IMT of the stomach in adults

No.	Author	Year	Age	Sex	Maximum tumor size (cm)	Preoperative diagnosis	Treatment	ALK immunostaining	IgG4/IgG ratio	Observation period	Recurrence
1	Kim et al. [4]	2004	26	M	8.0	N.D	TG, DP, SP, TC	N.D	N.D	5 weeks	Peritoneal dissemination recurrence
2	Leon et al. [5]	2006	50	F	7.0	N.D	DG	N.D	N.D	2 years	None
3	Park et al. [6]	2008	55	F	8.5	N.D	PR	Negative	N.D	N.D	N.D
4	Shah et al. [7]	2008	80	F	1.5	N.D	EMR	N.D	N.D	N.D	N.D
5	Shi et al. [8]	2010	40	F	5.8	N.D	PR	Positive	N.D	4 years	None
6	Shi et al. [8]	2010	45	M	5.5	N.D	PR	Positive	N.D	2.6 years	None
7	Shi et al. [8]	2010	40	M	6.3	N.D	PR	Positive	N.D	3.3 years	None
8	Shi et al. [8]	2010	42	M	8.0	N.D	PR	Positive	N.D	2 years	Local recurrence at 12 months
9	Shi et al. [8]	2010	36	M	4.5	N.D	PR	Positive	N.D	5 years	None
10	Albayrak et al. [9]	2010	56	F	6.0	N.D	PR	Negative	N.D	8 months	None
11	Fong et al. [10]	2010	56	M	3.0	N.D	PR	N.D	N.D	N.D	N.D
12	Lee et al. [11]	2011	42	F	5.5	GIST susp.	Endoscopic local tumor excision	N.D	N.D	N.D	N.D
13	Riberio et al. [12]	2012	37	F	7.3	GIST susp.	DG	Positive	N.D	N.D	None
14	Jain et al. [13]	2012	35	F	1.1	N.D	PR	Negative	N.D	7 months	None
15	Bijelovic et al. [14]	2013	43	F	2.5	N.D	DG	Positive	N.D	24 months	None
16	Arslan et al. [15]	2013	65	F	11.4	N.D	PR	Positive	N.D	37 months	None
17	Chong et al. [16]	2013	59	M	10.0	N.D	PR	Negative	N.D	N.D	N.D
18	Katakwar et al. [17]	2014	45	M	5.7	GIST susp.	DG	Positive	N.D	N.D	N.D
19	Chen et al. [18]	2014	50	F	22.0	N.D	TG, SP	Negative	N.D	4 months	N.D
20	Kim et al. [19]	2015	25	M	4.0	N.D	LPG	Negative	< 0.4	18 months	None
21	Mohammad et al. [20]	2016	18	M	10.0	Submucosal tumor susp.	LPG, SP	Negative	N.D	9 months	None
22	Jadhav et al. [21]	2017	18	M	8.6	Gastric leiomyoma, GIST susp.	PR	Negative	N.D	5 years	None
23	Ning et al. [22]	2017	50	F	3.0	Ectopic pancreas	ESD	N.D	N.D	2 years	None
24	Fan et al. [23]	2017	37	M	4.5	GIST susp.	TG	Positive	< 0.4	6 months	None
25	Lee et al. [24]	2018	35	F	2.5	N.D	PR	N.D	N.D	3 years	None
26	Lee et al. [24]	2018	39	F	2.0	N.D	PR	N.D	N.D	3 years	None
27	Lee et al. [24]	2018	38	F	4.2	N.D	PR	N.D	N.D	4 years	None
28	Lee et al. [24]	2018	36	F	3.0	N.D	PR	N.D	N.D	6 years	None
29	Lee et al. [24]	2018	43	F	3.4	N.D	PR	N.D	N.D	11 years	None
30	Hayashi et al. [25]	2018	53	F	2.0	Scirrhous gastric cancer susp.	PR(combined laparoscopic and endoscopic method)	Positive	N.D	N.D	N.D
31	Song et al. [26]	2020	43	M	4.5	N.D	DG	N.D	N.D	70 months	None
32	Hanjog et al. [27]	2021	25	F	10.0	GIST susp.	DG	N.D	N.D	6 months	None
33	Wang et al. [28]	2021	22	F	3.2	N.D	N.D	Positive	N.D	34 months	None
34	Wang et al. [28]	2021	41	M	6.1	N.D	N.D	Positive	N.D	12 months	None
35	Wang et al. [28]	2021	27	M	3.2	N.D	N.D	Positive	N.D	38 months	None
36	Flores-Trujillo et al. [29]	2022	52	F	7.6	Adenocarcinoma susp.	DG	Negative	N.D	N.D	None
37	Our case	2023	47	M	2.7	GIST susp.	PR(combined laparoscopic and endoscopic method)	Positive	> 0.5	17 months	None

N.D: Not documented; TG: Total gastrectomy; DG: Distal gastrectomy; PG: Proximal gastrectomy; PR: Partial resection; DP: Distal pancreatectomy; SP: Splenectomy; TC: Transverse colectomy; ESD: Endoscopic submucosal dissection; EMR: Endoscopic mucosal resection

IMT is generally diagnosed through histopathological examination. Typical histological features include infiltration of inflammatory cells, presence of eosinophils, and presence of spindle-shaped myofibroblastic cells. Immunohistochemical studies confirm the positivity of specific markers, such as SMA and ALK [3, 30]. In our case, immunostaining for SMA were positive in the proliferating spindle-shaped cells. The efficacy of ALK immunostaining has also been reported because immunopositivity for ALK and specific findings of the ALK gene locus in fluorescence in situ hybridization (FISH) testing are known to be specific to IMT [3]. In previous cases with IMT of the stomach in which immunostaining was performed, ALK positivity was confirmed in 40.5% (15/37) of cases (Table 1). It is recommended that ALK immunostaining and FISH testing be performed as much as possible as genetic analyses to obtain a diagnosis in cases of suspected IMT [3]. None of the cases were diagnosed before the surgery (Table 1), which indicates the difficulty of diagnosing IMT before surgery. Therefore, this disease may also be considered if a preoperative histological diagnosis cannot be made.

In the diagnosis of IMT, it is crucial to differentiate IMT from IgG4-related disease (IgG4-RD) because of their pathological similarities, including inflammatory cell infiltration and high number of IgG4-positive cells [31, 32]. While the relationship between IMT and IgG4-RD is not fully understood, a recent report has indicated that IMT can also exhibit IgG4-positive lymphocyte infiltration, emphasizing the need for differentiation from IgG4-RD [33]. Confirmation of the diagnosis can be achieved through immunohistochemical staining for ALK, which is specific and positive in the nuclei of IMT tumor cells. The absence of elevated serum IgG4 levels and multiple sclerotic tumor lesions also aids in differentiating IMT from IgG4-RD. Treatment options differ greatly between these two conditions, as inflammatory pseudotumors of IgG4-RD require steroids or immunosuppressive agents, whereas IMT is classified as benign or malignant based on various factors [34, 35]. On the other hand, IMT is classified as benign or malignant based on the shape of the tumor cells, the proliferation index, the number of mitotic figures, and the presence of necrosis, according to the WHO classification in 2020 [36]. The local recurrence rate is reported to be approximately 25% and distant metastasis is rare in all abdominopelvic organs [3]. Thus, differentiating between the diagnoses is critical, and ALK expression is considered a crucial diagnostic marker for this purpose. Interestingly, in this case, the expression of IgG4 was observed in > 50% of the cells, unlike in previous cases. Therefore, the accumulation of similar cases is needed in the future to determine their characteristics and prognosis.

Partial resection was performed in 54.3% (19/35) of the patients who underwent surgery. Among these cases with partial resection, local recurrence occurred in 5.3% (1/19), which is lower than the recurrence rate of resected IMT cases in all abdominopelvic organs because of the local recurrence rate is reported to be approximately 25% [3]. Therefore, if appropriate margins can be ensured, partial resection can be expected to preserve the stomach while achieving curative resection, and may be acceptable. Regarding the operative procedure, we selected for LECS, which allows for laparoscopic and endoscopic observation to ensure a secure margin and prevent dissemination with consideration of the preoperative diagnosis of KIT-negative GIST without ulceration. Classical LECS has been criticized for the potential problem of tumor exposure within the abdominal cavity and leakage of gastric contents during surgery [37]. However, inverted LECS overcomes this issue by supporting the entire gastric wall containing the tumor with sutures, minimizing the risk of gastric content leakage, and avoiding contact with other organs. Furthermore, inverted LECS is also valuable for making a diagnosis. Like our case, complete en bloc resection of the lesion is necessary for SMTs when the diagnosis is challenging and the possibility of malignancy cannot be ruled out. Inverted LECS offers the advantages of relatively straightforward surgical procedures, a reduced risk of intraperitoneal dissemination, and high diagnostic utility. Particularly for tumors located in difficult-to-reach areas of the stomach, such as the posterior wall or the greater curvature side, the use of supporting sutures for elevation and inversion of the stomach proved to be valuable. Hayashi et al. [25] also reported the efficacy of LECS, with no recurrence was observed during their follow-up period. Therefore, LECS, which allows the visualization of the margins from both inside and outside the stomach, may be considered as a surgical option. Other operative procedures such as non-exposure techniques to prevent iatrogenic intraperitoneal dissemination (e.g., CLEAN-NET LECS [38] and non-exposed endoscopic wall-inversion surgery (NEWS) [39]), have also been recently introduced. Inverted LECS is a relatively simple procedure in comparison to these methods. However, these procedures could be safer in consideration of oncological manipulation. Therefore, CLEAN-NET LECS and NEWS should be also considered in similar situations in the future.

## Conclusion

We encountered a rare case of IMT of the stomach that was successfully treated using inverted laparoscopic and endoscopic cooperative surgery. The diagnosis of this condition may be difficult both before and after surgery. In addition, it was challenging to differentiate between IgG4-RD and IMT based on pathological findings. When encountering a submucosal tumor originating in the stomach, the possibility of IMT should be considered, and efforts should be made to achieve complete tumor resection, while ensuring a definitive diagnosis through immunostaining and FISH analysis.

## Data Availability

Not applicable.

## Abbreviations

IMT: Inflammatory myofibroblastic tumor
LECS: Laparoscopic and endoscopic cooperative surgery
GIST: Gastrointestinal stromal tumor
EVG: Elastica van Gieson
FISH: Fluorescence in situ hybridization
NEWS: Non-exposed endoscopic wall-inversion surgery
